# Nanoparticles and Their Biological Applications: Recent Advances in 2022–2023

**DOI:** 10.3390/microorganisms12030489

**Published:** 2024-02-28

**Authors:** Wajid Zaman, Hakim Manghwar

**Affiliations:** 1 Department of Life Sciences, Yeungnam University, Gyeongsan 38541, Republic of Korea; 2 Lushan Botanical Garden, Jiangxi Province and Chinese Academy of Sciences, Jiujiang 332000, China

This Special Issue illustrates the collaborative effort required to harness the potential of nanoparticles, showcasing their role in targeting drug-resistant bacteria and highlighting new pathways for drug delivery, diagnostics, and beyond. Each article contributes advanced knowledge to our understanding of nanoparticle synthesis, functionalization, and its numerous applications. Recent studies delve into the practical implications of nanoparticles in combating antimicrobial resistance (AMR), where traditional antibiotics are increasingly ineffective [1,2,3]. Nanoparticles emerge as a beacon of hope, offering new mechanisms to kill pathogens and mitigate their uncontrollable effects through conventional methods [4,5,6].

This Special Issue notably addresses the potential of nanoparticles to offer new mechanisms against pathogen resistance compared to conventional treatments, revealing innovative strategies that could lead to more effective therapies. Furthermore, it explores the bio-formulation of nanoparticles, emphasizing the importance of eco-friendly methods, and the synergistic effects of nanoparticles and plant metabolites in scientific research and its application. A significant emphasis is placed on green chemistry approaches to nanoparticle synthesis, reflecting a growing acknowledgment of the need for sustainability in scientific research. These methods not only reduce environmental impacts but also enhance the antimicrobial properties of nanoparticles, presenting a win–win scenario for both healthcare and environmental conservation [7,8].

Despite the promising advancements, this editorial also acknowledges future challenges, including the toxicity of nanoparticles and their long-term environmental impacts. It highlights the necessity for ongoing research to understand and mitigate potential risks associated with widespread nanoparticle use. This Special Issue serves as a base for sharing knowledge and spurring further research in the field of green nanotechnology. It is a call to action for scientists, healthcare professionals, microbiologists, and chemists to integrate the basic principles of their fields to advance the development and application of nanoparticles. The goal is clear: to outmaneuver antimicrobial resistance and other biological challenges with the help of bio-manufactured nanoparticles.

In conclusion, this Special Issue, “Nanoparticles and Their Biological Applications: Recent Advances in 2022–2023”, provides valuable insights into the recent advancements and rising challenges in the field of antimicrobial nanoparticles. It highlights the significant potential of biosynthesized nanoparticles in addressing microbial resistance, offering insights into innovative strategies for developing new antimicrobial agents. As the field evolves, the contributions within this Special Issue pave the way for practical applications in the synthesis of nanoparticles, microbial resistance, healthcare, agriculture, and environmental protection, marking significant steps toward overcoming the most daunting challenges in modern science.

## List of Contributions

As we reflect on the contributions of this Special Issue, it is evident that the exploration of nanoparticles and their biological applications is a field with significant contributions to the field of microbiology, healthcare, and agriculture. The collaborative efforts of scientists across various disciplines will undoubtedly continue to drive this field forward, unraveling new knowledge and developing technologies that could revolutionize our approach to health, medicine, and environmental stewardship [9,10,11]. This Special Issue, “Nanoparticles and Their Biological Applications: Recent Advances in 2022–2023”, not only provides an understanding of the current state of research but also sets the stage for the next wave of innovations in antimicrobial strategies, urging the scientific community to pursue further knowledge and applications in this field. This collection, featuring nine articles, spans a broad spectrum of groundbreaking research. It not only highlights the versatility of nanoparticle synthesis methods and biological applications but also sets a foundation for future explorations in nanotechnology. These studies collectively advance our understanding of nanoparticle synthesis, functionalization, and their diverse applications, ranging from healthcare innovations to environmental sustainability efforts.

1. Al-Otibi, F.O.; Yassin, M.T.; Al-Askar, A.A.; Maniah, K. Green Biofabrication of Silver Nanoparticles of Potential Synergistic Activity with Antibacterial and Antifungal Agents against Some Nosocomial Pathogens. *Microorganisms* **2023**, *11*, 945. https://doi.org/10.3390/microorganisms11040945.
2. Al-Zaban, M.I.; Alhag, S.K.; Dablool, A.S.; Ahmed, A.E.; Alghamdi, S.; Ali, B.; Al-Saeed, F.A.; Saleem, M.H.; Poczai, P. Manufactured Nano-Objects Confer Viral Protection against Cucurbit Chlorotic Yellows Virus (CCYV) Infecting *Nicotiana benthamiana*. *Microorganisms* **2022**, *10*, 1837. https://doi.org/10.3390/microorganisms10091837.
3. Almaary, K.S.; Yassin, M.T.; Elgorban, A.M.; Al-Otibi, F.O.; Al-Askar, A.A.; Maniah, K. Synergistic Antibacterial Proficiency of Green Bioformulated Zinc Oxide Nanoparticles with Potential Fosfomycin Synergism against Nosocomial Bacterial Pathogens. *Microorganisms* **2023**, *11*, 645. https://doi.org/10.3390/microorganisms11030645.
4. Ansari, M.; Ahmed, S.; Abbasi, A.; Hamad, N.A.; Ali, H.M.; Khan, M.T.; Haq, I.U.; Zaman, Q.U. Green Synthesized Silver Nanoparticles: A Novel Approach for the Enhanced Growth and Yield of Tomato against Early Blight Disease. *Microorganisms* **2023**, *11*, 886. https://doi.org/10.3390/microorganisms11040886.
5. Farooq, A.; Khan, U.A.; Ali, H.; Sathish, M.; Naqvi, S.A.; Iqbal, S.; Ali, H.; Mubeen, I.; Amir, M.B.; Mosa, W.F.A.; et al. Green Chemistry Based Synthesis of Zinc Oxide Nanoparticles Using Plant Derivatives of *Calotropis gigantea* (Giant Milkweed) and Its Biological Applications against Various Bacterial and Fungal Pathogens. *Microorganisms* **2022**, *10*, 2195. https://doi.org/10.3390/microorganisms10112195.
6. Felifel, N.T.; Sliem, M.A.; Kamel, Z.; Bojarska, J.; Seadawy, M.G.; Amin, R.M.; Elnagdy, S.M. Antimicrobial Photodynamic Therapy against *Escherichia coli* and *Staphylococcus aureus* Using Nanoemulsion-Encapsulated Zinc Phthalocyanine. *Microorganisms* **2023**, *11*, 1143. https://doi.org/10.3390/microorganisms11051143.
7. Kim, S.-M.; Choi, H.-J.; Lim, J.-A.; Woo, M.-A.; Chang, H.-J.; Lee, N.; Lim, M.-C. Biosynthesis of Silver Nanoparticles from *Duchesnea indica* Extracts Using Different Solvents and Their Antibacterial Activity. *Microorganisms* **2023**, *11*, 1539. https://doi.org/10.3390/microorganisms11061539.
8. Srichaiyapol, O.; Maddocks, S.E.; Thammawithan, S.; Daduang, S.; Klaynongsruang, S.; Patramanon, R. TA-AgNPs/Alginate Hydrogel and Its Potential Application as a Promising Antibiofilm Material against Polymicrobial Wound Biofilms Using a Unique Biofilm Flow Model. *Microorganisms* **2022**, *10*, 2279. https://doi.org/10.3390/microorganisms10112279.
9. Ullah, Z.; Gul, F.; Iqbal, J.; Abbasi, B.A.; Kanwal, S.; Chalgham, W.; El-Sheikh, M.A.; Diltemiz, S.E.; Mahmood, T. Biogenic Synthesis of Multifunctional Silver Oxide Nanoparticles (Ag_2_ONPs) Using *Parieteria alsinaefolia* Delile Aqueous Extract and Assessment of Their Diverse Biological Applications. *Microorganisms* **2023**, *11*, 1069. https://doi.org/10.3390/microorganisms11041069.
